# Inflammatory Pathways in Coronary Artery Disease: Which Ones to Target for Secondary Prevention?

**DOI:** 10.3390/cells14030153

**Published:** 2025-01-21

**Authors:** Wan-Hei Cheng, Ying Wang

**Affiliations:** 1 Department of Pathology and Laboratory Medicine, Faculty of Medicine, University of British Columbia, Vancouver, BC V6T 1Z7, Canada; melody.cheng@hli.ubc.ca; 2 Centre for Heart Lung Innovation, University of British Columbia, Vancouver, BC V6Z 1Y6, Canada

**Keywords:** coronary artery disease, secondary prevention, inflammation

## Abstract

Coronary artery disease (CAD), the build-up of atherosclerotic plaques on the wall of blood vessels, causes adverse cardiovascular events. Secondary prevention focuses on treating patients with existing plaques to prevent disease progression. Recent studies have shown that inflammation is an independent risk factor that drives disease progression, and targeting inflammation could be an effective therapeutic strategy for secondary prevention. In this review, we highlighted the roles of several inflammatory pathways in rupture and erosion, two major processes through which established plaques lead to adverse cardiovascular events. In the past 15 years, numerous clinical trials have tested the therapeutic potential of targeting these pathways, including neutralizing inflammatory cytokines and blocking signaling transduction of the inflammatory pathways. Only colchicine was approved for clinical use in patients with CAD. This is primarily due to the multifaceted roles of inflammatory pathways in disease progression. Commonly used pre-clinical models provided robust information for the onset of early disease but limited understanding of the inflammatory network in established plaques. This review will summarize lessons learned from successful and failed clinical trials to advocate for assessing inflammation in established plaques before designing therapeutics for secondary prevention.

## 1. Introduction

**Figure 1:**
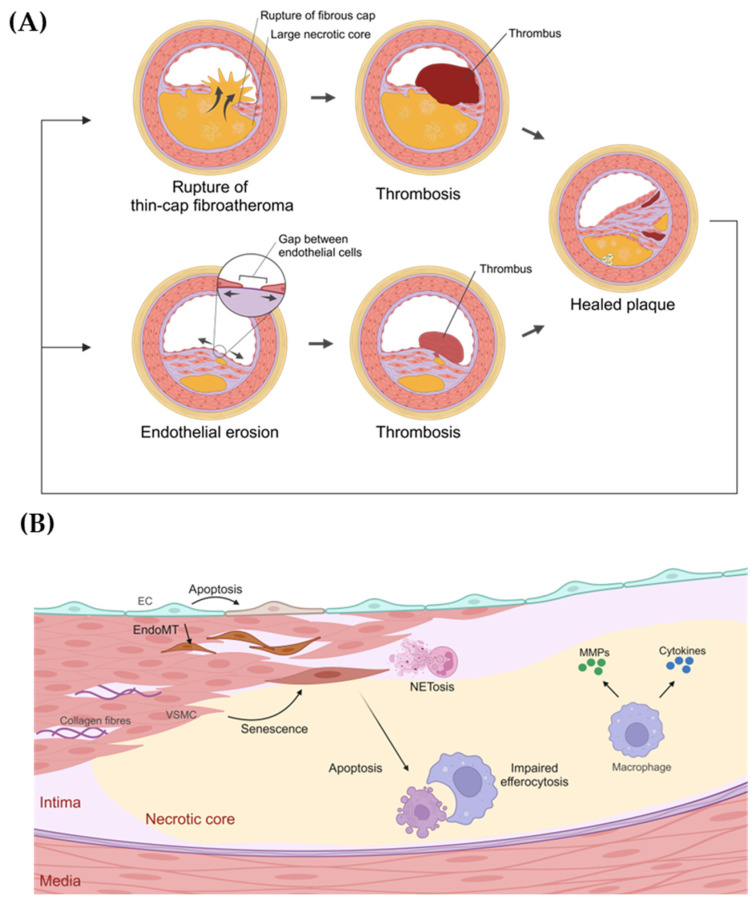
Established plaques prone to thrombosis. (**A**) Rupture of the fibrous cap and endothelial erosion are the most common causes of coronary thrombosis. (**B**) Changes in plaque cells that may contribute to rupture or erosion of established plaques. EC: endothelial cell; EndoMT: endothelial-to-mesenchymal transition; VSMC: vascular smooth muscle cell; NETosis: neutrophil extrusion of neutrophil extracellular traps (NETs); MMPs: matrix metalloproteinases.

Coronary artery disease (CAD) is the world’s leading cause of death, accounting for one-sixth of the total deaths worldwide, or 9.1 million lives every year [1]. CAD occurs when plaques build up in the coronary arteries in a process called atherosclerosis [2]. As this process is asymptomatic at its early stages [3], by the time patients develop symptoms and are diagnosed, the plaques are usually well-developed in the coronary arteries, restricting the flow of blood to the cardiac muscles [2]. Pathology studies of established plaques that led to acute coronary thrombosis revealed three types of structural changes: rupture of the fibrous cap (in 55–65% of cases), endothelial erosion (30–35% of cases), or the formation of calcified nodules (2–7% of cases) [4,5]. Rupture of the fibrous cap occurs in a type of established plaque called thin-cap fibroatheroma [3]. These plaques have large necrotic cores covered by thin fibrous caps, and the necrotic cores are often highly inflamed [3]. Upon rupture, the exposure of the necrotic core to the flowing blood triggers thrombosis. (Figure 1A) [3,6]. On the other hand, erosion occurs in plaques with thick fibrous caps or plaques devoid of necrotic cores [3]. These plaques are less inflamed, but the loss of the endothelial layer leads to thrombosis in the eroded area (Figure 1A) [3,6]. After thrombotic events, plaques may heal by developing thick fibrous caps [3], which further narrow the lumen of blood vessels and may again rupture or erode (Figure 1A) [3]. It is estimated that more than 300 million people globally are living with these “high-risk” established plaques [7]. A large, vulnerable patient population requires effective secondary prevention to inhibit the progression of established plaques and to reduce future risk of cardiovascular events.

Statins, a group of cholesterol-lowering drugs, are routinely used in patients with CAD. However, normalizing cholesterol with statins only lowers the relative risk of cardiovascular events by 30% [8]. Recent studies have identified chronic inflammation in established plaques as another independent risk factor. Anti-inflammatory therapeutics may, therefore, be of use in secondary prevention. The success of the LoDoCo (Low-Dose Colchicine) trial is the best evidence: in this trial, a low dose of colchicine, an anti-inflammatory drug that blocks microtubule assembly, reduced the risk of cardiovascular events in patients with stable CAD [9]. However, many other anti-inflammatory drugs failed to improve cardiovascular outcomes, such as the use of the p38α/β mitogen-activated protein kinase (p38 MAPK) inhibitor losmapimod in patients with a history of acute myocardial infarction (MI) [10]. Results from clinical trials suggest that not all inflammatory pathways can serve as therapeutic targets for secondary prevention, and it is crucial to understand what inflammatory pathways in established plaques contribute to rupture or erosion [11].

This review aims to provide a comprehensive overview of how different inflammatory pathways may contribute to the progression of established plaques and what should be taken into consideration when selecting them as targets for secondary prevention. The pitfalls of using cell culture and animal models to select therapeutic targets are discussed, which may explain why some of these anti-inflammatory drugs have failed to demonstrate beneficial effects in clinical trials.

## 2. Cells in Established Atherosclerotic Plaques

In established atherosclerotic plaques that are prone to rupture or erosion, the main cell types are endothelial cells (ECs), vascular smooth muscle cells (VSMCs), macrophages, and neutrophils (Figure 1B). Single-cell RNA sequencing (scRNA-seq) has shown that each of these cell types has various phenotypes in atherosclerotic plaques [12,13]. As the specific function of each phenotype is not well understood, we focus on the general functional changes in each cell type that may lead to rupture or erosion.

### 2.1. Endothelial Cells (ECs)

In established plaques, ECs form a continuous barrier between the blood flow and pro-thrombotic contents, such as the necrotic core and collagen [14]. Apoptosis and endothelial-to-mesenchymal transition (EndoMT) impair the integrity of ECs and contribute to plaque rupture or erosion.

Apoptosis: Increased apoptosis is detected in the ECs of human carotid plaques [15]. In vitro and in vivo studies have shown that ECs in established plaques are exposed to excess oxidized low-density lipoprotein (oxLDL) [16], hypoxia [17], tumor necrosis factor-α (TNF-α) [18], low shear stress [19,20], and oscillatory blood flow [21], all of which can trigger apoptosis. In vitro, apoptotic ECs release endothelial microparticles (EMPs) [22], which trigger thrombosis when injected into rats [23]. Increased EMPs were observed in the blood of patients with acute coronary syndrome (ACS) compared to patients without CAD [24,25], suggesting that apoptosis of ECs may promote thrombosis in established plaques.

Endothelial-to-mesenchymal transition (EndoMT): EndoMT is a biological process in which ECs acquire a mesenchymal cell phenotype [26]. It may involve three steps: (1) Loosened cell junctions between ECs allow their detachment from the monolayer on the apical side of the vascular wall; (2) increased motility enables ECs to protrude across the basement membrane into the fibrous cap enriched with VSMCs (Figure 1B); and (3) phenotypic changes in ECs increase their expression of mesenchymal cell markers, such as ACTA2, that are commonly expressed by VSMCs [26]. Using an EC-lineage tracing *ApoE*^−/−^ mouse model of atherosclerosis, increased EndoMT was observed as the disease progressed from early to advanced stage [27]. Around 20% of the ACTA2^+^ cells in the fibrous cap of fibroatheroma were derived from ECs in mice, indicating the contribution of EndoMT to fibrous cap formation [28]. EndoMT dramatically increased when VSMCs were depleted from the fibrous cap [28], suggesting that EndoMT was activated to maintain the structural integrity of fibrous caps, compensating for the loss of VSMCs [26]. However, fibrous caps enriched with ECs that have undergone EndoMT lacked collagen content and stability [28]. EndoMT was negatively correlated with the thickness of the fibrous cap in human plaques, and more EndoMT was seen in ruptured plaques compared to non-ruptured plaques [27]. Currently, EndoMT is considered a maladaptation that contributes to both plaque rupture and erosion [26,29], although the mechanism remains to be fully understood.

### 2.2. Vascular Smooth Muscle Cells (VSMCs)

VSMCs in established plaques play a crucial role in maintaining structural stability, and most previous studies correlated their functional changes to plaque rupture. VSMCs are the predominant cell type and the main source of collagen in the fibrous caps [30]. A total of 95% of ruptured coronary plaques contain thin fibrous caps (<65 μm) that lack VSMCs and collagen contents, suggesting that VSMCs in this region protect fibroatheroma from rupture [31]. ScRNA-seq of human carotid plaques has identified various VSMC phenotypes, including myofibroblast-like, foam cell-like, and pro-inflammatory subtypes, all of which contribute to the remodeling of the extracellular matrix (ECM) and plaque stability [32]. We focus on how apoptosis and senescence of VSMCs lead to the enlargement of the necrotic core and thinning of the fibrous cap [33], two morphological features that make established plaques prone to rupture.

Apoptosis: In humans, apoptotic VSMCs were found in the necrotic core beneath the rupture site and in shoulder regions where it is prone to rupture [34,35]. Apoptotic VSMCs are a signature of advanced plaques [35,36]. In advanced plaques of *ApoE*^−/−^ mice, induced VSMC apoptosis in the fibrous cap is associated with the loss of collagen, thinning of the fibrous cap, and enlargement of the necrotic core [37,38]. Bauriedel et al. found an increase in VSMC apoptosis in patients with unstable angina compared to those with stable CAD [39]. Combined, these observations indicate the association of apoptotic VSMCs with plaque destabilization and plaque rupture. Accumulated apoptotic VSMCs could be attributed to excess lipids and impaired efferocytosis in advanced plaques [33,40]. VSMCs make up at least half of the foam cells in coronary plaques, and these foam cells may undergo apoptosis upon lipid overload [41,42]. Apoptotic cells are normally cleared by phagocytes in a process called efferocytosis. However, this process is impaired in advanced plaques in both animal models and humans [40], which leads to secondary necrosis and the formation of necrotic cores [43]. In vitro, necrosis of apoptotic VSMCs resulted in the release of interleukin 1 alpha (IL-1α) and beta (IL-1β) [44], which contribute to inflammation and enlargement of the necrotic core in rupture-prone plaques.

Senescence: In addition to apoptosis, senescence, in which cells irreversibly stop proliferating, was observed in the fibrous caps of human carotid plaques [45]. The senescence of VSMCs has been linked to telomere shortening in chromosomes due to over-proliferation [46]. Inducing a mutation in a telomere-protecting protein resulted in VSMC senescence and thinning of the fibrous cap in *ApoE*^−/−^ mice [47]. Senescent VSMCs secrete pro-inflammatory cytokines, such as IL-1, IL-6, and matrix metalloproteinases (MMPs), which contribute to chronic inflammation [33]. More studies are being conducted to investigate the mechanisms of VSMC senescence and their contributions to plaque rupture or erosion.

### 2.3. Neutrophils

Although the number of neutrophils is relatively low compared to other cell types in established plaques, growing evidence has found a positive correlation between neutrophils and coronary thrombosis [48]. An autopsy study found increased neutrophil infiltration in ruptured and eroded plaques compared to other advanced plaques [49]. Recent studies have focused on protein degranulation and neutrophil extracellular traps (NETs) to investigate the potential mechanism underlying neutrophil-mediated plaque rupture and erosion [50].

Neutrophil degranulation: Numerous proteins are stored inside the granules of neutrophils, including α-defensins, neutrophil elastase, and MMP-8 [50]. In vitro studies have found that α-defensins increase platelet aggregation and thrombus formation [51], neutrophil elastase induces apoptosis of ECs [52], and MMP-8 breaks down collagens [53]. These are the potential mechanisms of how proteins released by neutrophil degranulation could contribute to thrombosis and rupture of established plaques in vivo. Indeed, increased neutrophil elastase and MMP-8 expression have been reported in rupture-prone and ruptured human plaques [52,54]. Notably, these proteins were traditionally believed to derive from neutrophils only [55,56], but recent studies reported their production in other plaque cells. Dollery et al. found that macrophages also produce neutrophil elastase [57]. VSMCs and macrophages can turn on MMP-8 expression during atherogenesis [55]. Therefore, it is difficult to directly link neutrophil degranulation to plaque rupture or thrombosis. Bulk RNA sequencing of human coronary plaques revealed that neutrophil degranulation is particularly enriched in patients with ischemic symptoms [58], suggesting a positive correlation between neutrophil degranulation and thrombotic events.

NETosis: Activated neutrophils in established plaques also play a role in thrombotic events by NETosis, a process to extrude the NET complexes made up of chromatin, granular proteins, and cytoplasmic proteins [59]. Increased NETosis was observed in ruptured plaques and eroded plaques compared to intact plaques in patients who died from MI [60]. NETosis-induced cell death is related to plaque rupture and erosion [61,62,63]. In human carotid arteries, NETs accumulate at the site of apoptotic ECs in erosion-prone plaques [61]. In vitro, the cytotoxic histone contents in NETs induced EC death [62]. Histone H4 derived from NETs also forms pores on VSMC membranes, which leads to necrosis and subsequent plaque instability in *ApoE*^−/−^ mice [63].

### 2.4. Macrophages

A large number of macrophages accumulate in established plaques, especially in rupture-prone regions [64]. In vitro, macrophages can be differentiated from monocytes into two extreme phenotypes: the M1-like subtype, which secretes pro-inflammatory cytokines, and the M2-like subtype, which promotes tissue repair [65,66]. In human and mouse plaques, macrophages have a spectrum of foamy, pro-inflammatory, and reparative phenotypes [12,13]. The role of macrophage apoptosis in necrotic core formation has been well-studied in the past 20 years [64,67]. Here, we focus on other functional changes that contribute to plaque rupture.

Impaired efferocytosis: Macrophages are the major source of phagocytes responsible for removing apoptotic cells via efferocytosis. The extensive death of macrophages in advanced plaques leads to the loss of functional phagocytes and impaired efferocytosis [68]. This further amplifies inflammation because functional phagocytes secrete anti-inflammatory cytokines following efferocytosis to prevent inflammation and secondary necrosis [69]. Impaired efferocytosis in advanced atherosclerosis is the result of reduced phagocytic activity of macrophages and the increased expression of “don’t eat me” molecules on apoptotic cells [69]. As discussed earlier, reduced efferocytosis capacity was detected in advanced carotid and coronary plaques [40,70]. Impaired efferocytosis results in the accumulation of apoptotic cells, which leads to enlarged necrotic cores and increased inflammation, promoting plaque rupture [14,71].

Secretion of MMPs: Pro-inflammatory macrophages produce various types of MMPs to break down the ECM, leading to thinning of the fibrous cap [65]. In vitro, increased mRNA expression of MMP-1, -2, -3, -7, -10, -14, and -25 was detected in pro-inflammatory M1-like macrophages [72]. Among these MMPs, MMP-1 and -13 are interstitial collagenases capable of breaking down collagens [73,74]. They are produced by cultured macrophages but not ECs or VSMCs in vitro [75], suggesting a unique role of macrophages in breaking down ECM in established plaques. In an ex vivo culture of fibrous caps dissected from human carotid plaques, adding macrophages increased collagen breakdown in the fibrous caps [76]. The breakdown was abolished by MMP inhibitors, confirming that macrophages break down the fibrous cap by secreting MMPs [76]. MMP-1- and MMP-13-positive macrophages co-localize with collagen degradation in rupture-prone plaques [75]. Combining this in vitro and ex vivo evidence, MMPs derived from macrophages play a key role in the rupture of established plaques.

In summary, the functional changes in plaque cells contribute to the progression of established plaques to rupture or erosion. One of the key drivers of their functional changes is inflammation.

## 3. Inflammatory Pathways in Atherogenesis

Pre-clinical studies have revealed several inflammatory pathways in the context of atherosclerosis. Some of these pathways are directly connected with functional changes, including cell death, NETosis, efferocytosis, and the production of MMPs and pro-inflammatory cytokines. These pathways can also shift the phenotypes of plaque cells to be more inflamed or increase the pro-inflammatory components, such as activated neutrophils and macrophages, in established plaques.

### 3.1. NOD-, LRR-, and Pyrin Domain-Containing Protein 3 (NLRP3) Inflammasome Pathway

The NLRP3 inflammasome pathway is one of the first active and well-characterized pro-inflammatory pathways found in established human plaques. It is commonly activated in various plaque cells, with macrophages and foam cells being more robust compared to other plaque cells [77]. NLRP3 inflammasome is highly expressed in unstable plaques compared to stable plaques [77]. In established plaques, stimuli such as TNF-α and cholesterol crystals activate the transcription factor nuclear factor κB (NF-κB) and upregulate the expression of components required to assemble the NLRP3 inflammasome in the priming step [78,79]. In the following activation step, these components oligomerize to form the NLRP3 inflammasome, which eventually leads to the cleavage of caspase-1 and the release of active pro-inflammatory cytokines IL-1β and IL-18 [79]. Notably, cellular microtubules are critical in this process, as they facilitate the assembly and activation of the NLRP3 inflammasome [80]. The disruption of microtubules suppresses the activation of the NLRP3 inflammasome in vitro [80]. Higher mRNA expression of NLRP3 and caspase-1 was detected in the carotid plaques of symptomatic patients compared to asymptomatic patients [81].

In cultured ECs and VSMCs, NLRP3 activation promotes pyroptosis, an inflammation-mediated cell death [82,83], contributing to endothelial erosion and thinning of the fibrous cap in established plaques. The activation of the NLRP3 inflammasome supports NETosis by promoting the breakdown of nuclear and plasma membranes to form NETs [84]. Inhibiting the NLRP3 inflammasome pathway led to reduced macrophages, MMP-2, and MMP-9 in *ApoE*^−/−^ mice, whereas the thickness of fibrous caps increased [85], suggesting that it is promising to stabilize atherosclerotic plaques by targeting the NLRP3 inflammasome pathway.

Results from these pre-clinical studies point in the same direction: the activated NLRP3 inflammasome pathway drives the progression of established plaques. The clear association with adverse outcomes makes NLRP3 inflammasome a promising therapeutic target for secondary prevention and inspired the CANTOS trial (Canakinumab Anti-Inflammatory Thrombosis Outcomes Study), which aims to reduce future cardiovascular events by neutralizing IL-1β.

### 3.2. Toll-like Receptor (TLR) Pathways

Toll-like receptors (TLRs) are transmembrane proteins widely expressed in many cell types, including vascular and immune cells [79]. Upon stimulation, the TLR pathway goes through either the classical myeloid differentiation primary response protein 88 (MyD88)-dependent pathway or the endosomal TIR domain-containing adaptor protein-inducing IFNβ (TRIF)-dependent pathway [79,86]. Both pathways eventually activate the mitogen-activated protein kinase (MAPK) and the NF-κB pathways to upregulate the expressions of pro-inflammatory cytokines and MMPs [79,86]. Various types of TLRs were observed in established human plaques, with TLR2 and TLR4 being studied the most in atherogenesis [87]. They are expressed by ECs, VSMCs, and macrophages [88].

In cultured plaque cells isolated from advanced plaques, anti-TLR2 antibodies reduced the expression of MCP-1, IL-8, and IL-6, whereas anti-TLR4 antibodies also reduced the expression of MMP-3 [87]. Cultured VSMCs isolated from *TLR4*-knockout mice had reduced production of IL-1β, TNF-α, MCP-1, and MMP-2 compared to wild-type mice [89]. These pro-inflammatory cytokines and MMPs promote inflammatory response and degradation of the ECM in established plaques.

Unlike the NLRP3 inflammasome pathway, the outcomes of inhibiting TLR-mediated inflammation using TLR antagonists have not been tested in patients with CAD. This is partially because blocking different TLRs expressed by various plaque cells may trigger diverse functional changes. Additionally, the long-term effects of TLR antagonists in the context of CAD have not been explored in pre-clinical studies to justify a clinical trial. Nevertheless, part of the anti-inflammatory effects of statins and colchicine is mediated by TLR pathways [90,91].

### 3.3. Complement Cascade

As part of innate immunity, the complement cascade plays a complex role in driving the progression of established plaques [92]. Activation of the complement cascade can be triggered by stimuli in established plaques, such as antibodies, cholesterol crystals, and C-reactive protein (CRP), via three different pathways: the classical, lectin, and alternative pathways [92]. Upon activation, the three pathways converge at the cleavage of complement component C3 into pro-inflammatory anaphylatoxin C3a and opsonin C3b [92]. C3b then binds to other complement components to form C5 convertase, resulting in the cleavage of C5 into C5a and C5b [92]. C5b ultimately induces the formation of the membrane attack complex (MAC), which forms pores on cell surfaces and causes cell lysis [92]. Human plaques highly express complement component proteins [93,94] and anaphylatoxin receptors C3aR and C5aR [95]. C3aR and C5aR were mainly found on macrophages, but they are also expressed on ECs, VSMCs, and T cells, suggesting that a broad plaque cell population can be affected by anaphylatoxins C3a and C5a [95]. C5a expression increased in unstable human coronary plaques compared to stable plaques [96], and its plasma level was found to be positively correlated with cardiovascular events in patients with advanced plaques [97]. In advanced human plaques, MAC was enriched in the deep intima [98], and it correlates with plaque instability [99] and cardiovascular events [100]. Combined, evidence from human biospecimens suggests that the cleavage of C5 is associated with the progression of established plaques to thrombosis.

Downstream of C5 cleavage, the complement cascade can drive the progression of established plaques via pro-inflammatory cytokines, MMPs, coagulation, and cell lysis. In cultured ECs, C5a induced the production of IL-8 and IL-1β [101]. Priming of monocytes with C5a amplified cholesterol crystals-activated expression of IL-1β [102]. Priming ex vivo cultured carotid plaques with C5a also increased the expression of IL-1β, IL-18 levels, and NLRP3 inflammasome by cholesterol [103]. Moreover, C5a also increased MMP-1 and -9 secretion from cultured macrophages [96]. C5a may promote thrombosis of established plaques, as suggested in cultured ECs [104]. In vivo, inhibiting MAC prevented apoptosis in advanced plaques of *ApoE*^−/−^ mice [105].

Notably, the complement cascade also plays a role in cell survival and efferocytosis in established plaques. It was suggested that when a limited amount of MAC is present on cell surfaces, the complement cascade may activate cell proliferation instead of causing cell lysis [106]. Pulanco et al. found that C1q, a complement protein in the classical pathway, enhanced the survival of oxLDL-loaded macrophages and the efferocytosis of apoptotic macrophages [107]. C3a was reported to promote macrophage phenotypic changes towards an anti-inflammatory subtype in vitro, and knocking out its receptor C3aR in *ApoE*^−/−^ mice led to an increase in pro-inflammatory M1-like macrophages in advanced plaques [108]. Taken together, these studies show the other side of the complement cascade that contributes to resolving inflammation in established plaques.

The complement cascade appears to play a multifaceted role in the progression of established plaques. About 50 components are involved in the network of the complement cascade, and they form multiple domains that affect different plaque cells. The integrated impact on established plaques could be dependent on the components of established plaques and the interplay between complement components in the plaques and in the circulation system. The integrated and context-specific effects need to be assessed before selecting one of the complement components as a therapeutic target for secondary prevention.

### 3.4. Janus Kinase 2/Signal Transducer and Activator of Transcription 3 (JAK2/STAT3) Pathway

Janus kinase 2 (JAK2) is a cytoplasmic tyrosine kinase attached to the intracellular side of transmembrane receptors [109]. Stimulated JAK2 activates signal transducer and activator of transcription 3 (STAT3) protein. Together, they translocate into the nucleus to regulate gene transcription [109,110]. The JAK2/STAT3 pathway is activated by various stimuli, including cytokines and oxLDL in established plaques [111,112]. STAT3 was directly involved in inflammation in the blood vessels [110]. Its increased activation was observed in advanced carotid plaques [113,114]. Similar to the complement cascade, the JAK2/STAT3 pathway could be a double-edged sword in atherosclerosis.

The well-known pro-inflammatory products of JAK2/STAT3 activation are oxidative stress and pro-inflammatory cytokines (e.g., IL-8, IL-18, and IL-6), as found in cultured VSMCs and ECs [114,115,116,117]. These pro-inflammatory products can induce cell death and contribute to the destabilization of established plaques. The conditional knockout of STAT3 in ECs reduced macrophage accumulation and plaque size in vivo [114]. The anti-inflammatory effects of the JAK2/STAT3 pathway in the context of atherosclerosis were also reported. Weekly intravenous injection of STAT3-carrying adenovirus reduced oxidative stress and macrophage accumulation in the vascular wall of *Ldlr*^−/−^ mice during early atherogenesis [118]. In vitro, statin inhibited hypoxia-induced apoptosis of ECs, and this effect depends on the activation of the JAK2/STAT3 pathway [119]. It was hypothesized that the dual role of the JAK2/STAT3 pathway in atherosclerosis depends on which isoform of STAT3 is activated: the pro-inflammatory STAT3α or the anti-inflammatory STAT3β [120]. However, these isoform-dependent effects have not been fully characterized.

In advanced human carotid plaques, Taleb et al. observed increased STAT3 activation in stable plaques with thick fibrous caps compared to unstable plaques [113]. Although it is difficult to define the contribution of the JAK2/STAT3 pathway to the formation of fibrous caps in vivo, we cannot rule out the possibility that its activation in some plaque cells and at certain stages of the disease is necessary to stabilize established plaques. Therefore, STAT3 itself has not been targeted for secondary prevention. Instead, a key product of the JAK2/STAT3 pathway, IL-6, was considered as a therapeutic target by several clinical trials.

## 4. Clinical Trials Using Anti-Inflammatory Drugs for Secondary Prevention of CAD

**Figure 2:**
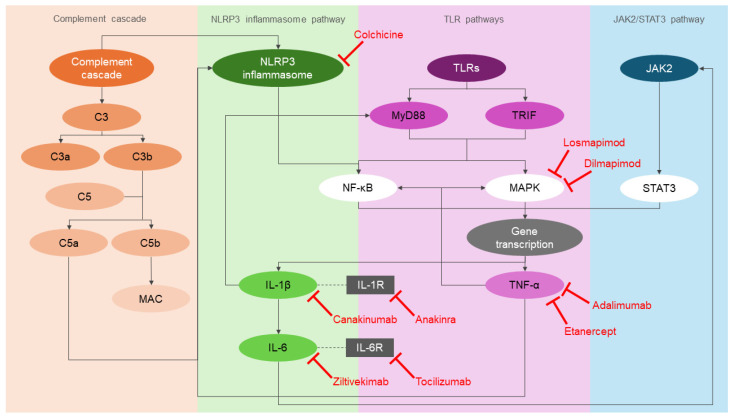
Overview of inflammatory pathways and their inhibitors. MAC: membrane attack complex; NLRP3: NOD-, LRR- and pyrin domain-containing protein 3; NF-κB: nuclear factor κB; IL-1β: interleukin 1 beta; IL-1R: interleukin 1 receptor; IL-6: interleukin 6; IL-6R: interleukin 6 receptor; TLRs: toll-like receptors; MyD88: myeloid differentiation primary response protein 88; TRIF: TIR domain-containing adaptor protein inducing IFNβ; MAPK: mitogen-activated protein kinases; TNF-α: tumor necrosis factor-α; JAK2: Janus kinase 2; STAT3: signal transducer and activator of transcription 3.

Growing evidence has shown that inflammation is a double-edged sword in atherogenesis. Several key proteins from the above inflammatory pathways have been chosen as therapeutic targets (Figure 2) to test the outcomes of anti-inflammatory drugs for secondary prevention of CAD.

### 4.1. IL-1 Signaling Pathway Inhibitors: Canakinumab and Anakinra

In the IL-1 family, IL-1α and IL-1β are pro-inflammatory interleukin 1 receptor 1 (IL-1R1) agonists, whereas IL-1Ra is an anti-inflammatory IL-1R1 antagonist [121]. Upon activation by either IL-1α or IL-1β, the intracellular domain of IL-1R1 binds to MyD88, which leads to the upregulation of pro-inflammatory genes (e.g., *IL-6* and *TNF-α*) through NF-κB [122,123]. Clinical trials have been performed to inhibit IL-1 signaling in patients with established coronary plaques. The strategies are to neutralize IL-1β or to prevent the binding of IL-1α and IL-1β with IL-1R.

Inspired by pre-clinical studies of the NLRP3 inflammasome pathway, the CANTOS trial (Canakinumab Anti-Inflammatory Thrombosis Outcomes Study) used a human monoclonal antibody (canakinumab) to neutralize IL-1β [124]. A total of 10,061 patients with a recent history of MI and CRP levels above 2 mg/L were recruited [122,124]. Canakinumab reduced the recurrence of cardiovascular events in these patients without lowering cholesterol levels [124]. However, the drug was associated with a risk of fatal infection [122]. Given the inconvenient administration method (injection) and the high cost of the antibody, the Food and Drug Administration (FDA) did not approve its clinical application as a secondary prevention for CAD [122]. Nevertheless, the success of the CANTOS trial provided evidence that inflammation is an independent risk factor for CAD [122].

Anakinra is a recombinant human IL-1Ra, which competes with IL-1α and IL-1β for binding to IL-1R1 and therefore inhibits their downstream signal transduction [123]. In the MRC-ILA Heart Study, anakinra reduced CRP levels but did not reduce adverse cardiovascular events in patients with ACS [125]. In the VCU-ART3 study (Virginia Commonwealth University Anakinra Remodeling Trial 3), anakinra reduced CRP levels, heart failure, hospitalization, and death in patients with MI [126]. Notably, as the maturation and secretion of IL-1α do not depend on cleaved caspase-1 in the NLRP3 inflammasome pathway [121,123], anakinra is expected to have a broader anti-inflammatory effect compared to canakinumab, raising concerns that it may mask the early signs of infection and delay timely antimicrobial treatment [127]. The ongoing VA-ART4 study (the Virginia–Anakinra Remodeling Trial 4) will investigate whether anakinra can prevent heart failure in patients while monitoring the side effects of the drug [128].

### 4.2. IL-6 Signaling Pathway Inhibitors: Tocilizumab and Ziltivekimab

Increased IL-6 levels were detected in CAD and found to be correlated with cardiovascular events [129]. IL-6 is therefore proposed to be a therapeutic target [130]. By binding to soluble or membrane-bound receptors on cell surfaces, IL-6 activates the JAK2/STAT3 pathway, which may have context-dependent effects in established plaques [131]. Of note, the binding of IL-6 to soluble receptors initiates trans-signaling, which exerts mainly pro-inflammatory effects and contributes to chronic inflammation [131,132]. Meanwhile, the binding of IL-6 to membrane-bound receptors initiates classic signaling, which is mainly responsible for the acute immune response. Tocilizumab and ziltivekimab are two antibodies that block both the trans- and the classic-signaling pathways.

Tocilizumab is a human monoclonal antibody that inhibits the effect of IL-6 by competitively binding to IL-6 receptors [133]. In patients with either type of ACS, non-ST-segment elevation MI (NSTEMI) [134] or ST-segment elevation MI (STEMI) [135], a single dose of tocilizumab reduced levels of CRP and myocardial damage. An ongoing phase II clinical trial, the DOBERMANN trial (Low-Dose Dobutamine and Single-Dose Tocilizumab in Acute Myocardial Infarction With High Risk of Cardiogenic Shock), will investigate tocilizumab’s effects in patients who underwent percutaneous coronary intervention (PCI) [136]. Tocilizumab was used as a single dose to tackle acute inflammation following MI and PCI. Long-term treatment of tocilizumab in patients with stable CAD, who have low and chronic inflammation, is debatable [133]. One of the concerns is elevated cholesterol levels, as observed in rheumatoid arthritis patients receiving a 3-month treatment of the drug [137].

Ziltivekimab is a new human monoclonal antibody that directly binds to IL-6 [133]. Its potential to reduce inflammation and improve cardiovascular outcomes is being tested in the ongoing ZEUS study (Ziltivekimab Cardiovascular Outcomes Study), which has enrolled patients with chronic kidney disease and established atherosclerotic plaques [138]. Other ongoing clinical trials [139,140] that will specifically test the cardiovascular outcomes of ziltivekimab are summarized in Table 1.

### 4.3. TNF-α Signaling Pathway Inhibitors: Etanercept and Adalimumab

TNF-α is a pro-inflammatory cytokine that is found in the necrotic core and is associated with impaired efferocytosis in rupture-prone atherosclerotic plaques [154]. TNF-α binds to two types of TNF-α receptors: TNFR1, which is expressed by most cells, and TNFR2, which is expressed by endothelial cells and immune cells [155]. The binding of TNF-α to TNFR1 activates the production of pro-inflammatory cytokines through p38 MAPK [155]; and the binding of TNF-α to TNFR2 activates the NF-κB pathway or the phosphatidylinositol-3-kinase signaling pathway, resulting in the promotion of cell proliferation, survival, and immunoregulatory responses [155]. Knocking out TNF-α in *ApoE*^−/−^ mice reduced plaque size [156].

Etanercept is a decoy receptor protein that binds to TNF-α with a high affinity [157]. It was used to treat various inflammatory diseases such as rheumatoid arthritis [157]. However, two clinical trials in heart failure patients did not observe any cardiovascular benefits of etanercept (Table 1) [141].

Adalimumab is a humanized monoclonal antibody that binds to and neutralizes TNF-α [158]. In patients with chronic inflammatory disease and having a high risk of cardiovascular disease, adalimumab reduces systemic inflammation [142,143]. However, the long-term cardiovascular outcomes were not followed up in these trials. Combined, there is no convincing evidence that etanercept or adalimumab could lead to beneficial effects for the secondary prevention of CAD. This can be explained by a study of TNF-α-deficient *ApoE*^−/−^ mice, which showed less necrosis but more apoptosis in advanced plaques [159]. This effect was attributed to the loss of NF-κB activation by TNF-α and the downstream anti-apoptotic function [159]. Therefore, it is debatable whether inhibiting TNF-α would benefit patients with advanced plaques when efferocytosis is already impaired. No active clinical trials have been launched to further pursue the use of TNF-α inhibitors in CAD.

### 4.4. P38 MAPK Signaling Pathway Inhibitors: Losmapimod and Dilmapimod

As mentioned before, the p38 MAPK is downstream of TLR pathways. Upon activation by various stimuli, such as pro-inflammatory cytokines, oxLDL, and hypoxia in established plaques, p38 MAPK turns on transcription factors to regulate the production of pro-inflammatory cytokines such as IL-1, IL-6, and TNF-α [160]. Similar to TNF-α, the pro-inflammatory effects of p38 MAPK made it a promising target for secondary prevention a decade ago. However, no p38 MAPK inhibitors have demonstrated improved cardiovascular outcomes in patients with CAD.

Both losmapimod and dilmapimod are p38 MAPK inhibitors [160]. In several phase II clinical trials, they reduced vascular inflammation or systemic inflammation in patients with diagnosed atherosclerotic disease [144] and those who recently received PCI [146]. However, the long-term cardiovascular outcomes were not assessed. In patients with ACS, two clinical trials did not observe any cardiovascular benefits of losmapimod [10,145]. It was suggested that many alternative pro-inflammatory signaling pathways work in parallel with p38 MAPK, which may bypass the inhibition of p38 MAPK and reduce the potential efficacy of p38 MAPK inhibitors [161].

### 4.5. Colchicine

Colchicine is an anti-inflammatory drug widely used in gout patients [133]. It exerts its anti-inflammatory effects on multiple targets in established plaques [133]. By inhibiting microtubule polymerization, colchicine prevents the assembly of the NLRP3 inflammasome [80,147,148]. By inhibiting the Rho A/Rho-associated protein kinase (ROCK) pathway, colchicine inhibits ATP-induced release of IL-1β in cultured macrophages [149]. Additionally, colchicine also inhibits NETosis of neutrophils in patients with ACS [162].

In the LoDoCo (Low-Dose Colchicine) trial, colchicine reduced cardiovascular events in patients with stable CAD [9]. In the LoDoCo2 study, a daily low dose of colchicine reduced the risk of cardiovascular events in patients with chronic CAD [151]. Colchicine also reduced the risk of cardiovascular events in patients who recently had MI in the COLCOT (Colchicine Cardiovascular Outcomes Trial) study [150]. However, when colchicine was used to treat patients shortly after ACS, no significant effect was observed in the COPS (Colchicine in Patients With Acute Coronary Syndrome) trial [152]. It is possible that colchicine may not be able to tackle the high inflammation level shortly after ACS in the COPS trial [152]. However, in the absence of a biomarker to assess the level of baseline inflammation, we cannot prove this assumption. In the COPS trial, where a higher dose of colchicine was used compared to the COLCOT trial, a higher rate of all-cause mortality was observed, raising concerns over the safety of colchicine use at a high dose [152]. The ongoing CLEAR SYNERGY (OASIS 9) trial, which currently has recruited 7,062 post-MI patients, will focus on the effectiveness of daily low-dose colchicine for reducing cardiovascular events in these patients within 72 h of PCI [153].

Lessons learned from these successful and failed clinical trials suggest that understanding the full picture of the inflammatory network and the activation status of inflammatory pathways in patients is critical for choosing the right inflammatory target for secondary prevention. Inhibiting broadly can increase the risk of over-suppression of immune responses or off-target side effects. Inhibiting signaling cascades that have multiple transduction pathways, such as the classic and trans-signaling pathways of IL-6, will lead to pleiotropic effects. It was suggested that selective inhibition of IL-6 trans-signaling is promising for treating atherosclerotic patients with fewer side effects [132]. However, the assumption needs to be supported by a better characterization of the IL-6 signaling network. Inhibiting TNF-α and p38 MAPK makes sense when taking their pro-inflammatory effects into consideration, but whether their associated inflammatory pathways are active and essential for driving disease progression in patients with CAD is more important in therapeutic design.

## 5. Limitations of Previous Studies and Future Directions

Statins, one of the first-line medications for patients with CAD, have proven anti-inflammatory properties independent of their cholesterol-lowering effects [163,164,165]. Most patients in the above clinical trials have received statins in the past. Therefore, to best predict the effectiveness of anti-inflammatory drugs, the ideal models in pre-clinical studies should reflect the status of inflammatory pathways after statin treatment.

Current pre-clinical models of atherosclerosis, including cultured cells and rodents, are seldom treated by statins before anti-inflammatory drugs are tested. Moreover, while these models provide valuable insights into early atherogenesis, they have limitations in providing a full picture of the inflammatory network for identifying therapeutic targets in patients with CAD.

### 5.1. Cell Culture Models

Traditional cell lines of ECs, SMCs, and macrophages do not accurately represent the cell phenotypes in advanced atherosclerotic plaques in vivo. For example, multiple phenotypes of VSMCs exist in human plaques [12,32], but in vitro models typically study the contractile phenotype of VSMCs [166]. Studying individual cell lines separately also provides limited insight into the complex intercellular crosstalk, ECM interactions, and structural remodeling involved in human atherosclerosis [166,167]. Although co-culture, microfluidic systems, and 3D tissue cultures have been established to mimic the architecture of blood vessels, studies using these models primarily focused on early atherogenesis, such as cholesterol deposition and immune cell infiltration, whereas secondary prevention aims to treat patients with advanced CAD [166].

### 5.2. Mouse Models

*ApoE*^−/−^ and *Ldlr*^−/−^ mice are the most commonly used animal models of atherosclerosis [167]. However, these models do not fully represent the disease status of patients requiring secondary prevention [167]. First of all, most experiments administer anti-inflammatory therapies to these mice before atherosclerotic plaques develop, whereas secondary prevention in humans is given to patients who already have established atherosclerotic plaques. For example, a p38 inhibitor was given to *ApoE*^−/−^ mice at 8 weeks of age, when plaques had not yet formed in these mice [168]. Findings from such early-staged diseased models may not apply to patients with advanced plaques, as the active inflammatory pathways in early and advanced plaques are different. Moreover, advanced plaques in *ApoE*^−/−^ and *Ldlr*^−/−^ mice are less complex than those in humans, and they seldom progress to rupture or erosion [169]. Last but not least, high-fat diets are often used to induce atherosclerosis, resulting in extreme hypercholesterolemia [169]. These mice recapitulate patients with familial hyperlipidemia rather than patients with CAD, whose cholesterol levels are controlled by lipid-lowering therapies [169]. Of note, hyperlipidemia increases inflammation in atherosclerotic plaques. These limitations make it clear that promising anti-inflammatory drugs tested in animal models may not be efficient in human patients.

### 5.3. Human Biospecimens

With the above limitations of in vitro and in vivo models, rupture- or erosion-prone plaques from patients who have received statin treatment are better models to reveal which residual inflammatory pathways to target for secondary prevention. Bulk RNA sequencing allows for hypothesis-free profiling of the entire transcriptome in a high-throughput manner, offering a broad view of pathways that are involved in human plaques [170]. Applying this method to human carotid and coronary plaques, the Athero-Express study identified the association of increased neutrophil degranulation with ischemic symptoms [58]. However, studying the transcriptome alone does not confirm the activation of these pathways, as post-translational modifications, protein folding, and activations occur after mRNA translation, altering protein structure and function [171]. For example, the JAK2/STAT3 pathway is activated by the phosphorylation of STAT3 [172]. A multi-omics study demonstrated that only a small proportion (8 of 587) of upregulated genes identified by RNA sequencing was enriched at the protein level in calcified valves [173]. Thus, the mRNA levels of inflammatory genes do not always correlate with the activity of the inflammatory pathways.

### 5.4. Future Directions

Anti-inflammatory drugs can provide additional benefits only if they effectively inhibit the disease-driving factors in advanced plaques. Therefore, we advocate for the action of characterizing inflammation in the disease context to determine which pathways to target for secondary prevention. Future studies are needed to (1) characterize active inflammatory pathways in advanced human plaques and (2) understand how the inflammatory network contributes to plaque erosion and rupture. It is also important to develop accessible and non-invasive methods, such as blood biomarkers and medical imaging, to identify patients who would benefit from anti-inflammatory drugs.

## Figures and Tables

**Table 1:** Clinical trials of anti-inflammatory therapies for the secondary prevention of CAD.

Target	Drug/Compound Name	Clinical Trial	Study Population	Treatment Outcome
IL-1 signaling pathway	Canakinumab (Rejected by FDA)	CANTOS [124]	Patients with a history of MI and CRP levels > 2 mg/L	Canakinumab reduced inflammation and adverse cardiovascular events, but the risk of fatal infection and the high cost of clinical application outweighed the benefits.
	Anakinra (Phase II trial ongoing)	MRC-ILA Heart [125]	Patients with ACS (NSTEMI)	Anakinra reduced inflammation but not the risk of future adverse cardiovascular events.
		VCU-ART3 [126]	Patients with ACS (STEMI)	Anakinra reduces inflammation and adverse cardiovascular events.
		VA-ART4 [128]	Patients with ACS (STEMI)	Clinical trial ongoing.
IL-6 signaling pathway	Tocilizumab (Phase II trial ongoing)	A phase II clinical trial [134]	Patients with ACS (NSTEMI)	Tocilizumab reduced inflammation and myocardial damage but not the risk of future adverse cardiovascular events.
		ASSAIL-MI [135]	Patients with ACS (STEMI)	
		DOBERMANN [136]	Patients recently received PCI	Clinical trial ongoing.
	Ziltivekimab (Phase III trials ongoing)	ZEUS [138]	Chronic kidney disease patients with evidence of CAD	Clinical trial ongoing.
		SPIDER [139]	Patients with CAD and CRP levels > 2 mg/L	
		ARTEMIS [140]	Patients with ACS	
TNF-α signaling pathway	Etanercept	RECOVER [141]	Heart failure patients	Etanercept did not improve clinical outcomes.
		RENAISSANCE [141]		
	Adalimumab	A subclinical trial [142]	Rheumatoid arthritis patients	Adalimumab reduced inflammation. Changes in cardiovascular events were not assessed.
		PIONEER [143]	Hidradenitis suppurativa patients (patients with a high risk of cardiovascular disease)	
p38 MAPK signaling pathway	Losmapimod	A phase II clinical trial [144]	Patients with diagnosed atherosclerotic disease	Losmapimod reduced inflammation. Cardiovascular outcomes were not assessed.
		SOLSTICE [145]	Patients with ACS (NSTEMI)	Losmapimod reduced inflammation but not the risk of future adverse cardiovascular events.
		LATITUDE-TIMI 60 [10]	Patients with ACS	
	Dilmapimod	A phase II clinical trial [146]	Patients with CAD	Dilmapimod reduces inflammation caused by PCI.
NLRP3 inflammasome pathway [80,147,148], RhoA/ROCK pathway [149]	Colchicine (Phase III trial ongoing)	LoDoCo [9]	Patients with stable CAD	Colchicine reduced adverse cardiovascular events.
		COLCOT [150]	Patients with a history of MI in the previous 30 days	
		LoDoCo2 [151]	Patients with stable CAD	
		COPS [152]	Patients with ACS	No statistically significant benefits.
		CLEAR SYNERGY (OASIS 9) [153]	Patients with ACS (STEMI) who received PCI	Clinical trial ongoing.

## Data Availability

No new data were created or analyzed in this study. Data sharing is not applicable to this article.

## References

[1] British Heart Foundation Global Heart & Circulatory Diseases Factsheet. https://www.bhf.org.uk/-/media/files/for-professionals/research/heart-statistics/bhf-cvd-statistics-global-factsheet.pdf.

[2] Mir M.A., Dar M.A., Qadir A. (2024). Exploring the Landscape of Coronary Artery Disease: A Comprehensive Review. Am. J. Biomed. Pharm..

[3] Bentzon J.F., Otsuka F., Virmani R., Falk E. (2014). Mechanisms of plaque formation and rupture. Circ. Res..

[4] Virmani R., Kolodgie F.D., Burke A.P., Farb A., Schwartz S.M. (2000). Lessons from sudden coronary death: A comprehensive morphological classification scheme for atherosclerotic lesions. Arterioscler. Thromb. Vasc. Biol..

[5] Otsuka F., Yasuda S., Noguchi T., Ishibashi-Ueda H. (2016). Pathology of coronary atherosclerosis and thrombosis. Cardiovasc. Diagn. Ther..

[6] Luo X., Lv Y., Bai X., Qi J., Weng X., Liu S., Bao X., Jia H., Yu B. (2021). Plaque erosion: A distinctive pathological mechanism of acute coronary syndrome. Front. Cardiovasc. Med..

[7] Stark B., Johnson C., Roth G.A. (2024). Global prevalence of coronary artery disease: An update from the global burden of disease study. J. Am. Coll. Cardiol..

[8] Byrne P., Demasi M., Jones M., Smith S.M., O’Brien K.K., DuBroff R. (2022). Evaluating the association between low-density lipoprotein cholesterol reduction and relative and absolute effects of statin treatment: A systematic review and meta-analysis. JAMA Intern. Med..

[9] Nidorf S.M., Eikelboom J.W., Budgeon C.A., Thompson P.L. (2013). Low-dose colchicine for secondary prevention of cardiovascular disease. J. Am. Coll. Cardiol..

[10] O’Donoghue M.L., Glaser R., Cavender M.A., Aylward P.E., Bonaca M.P., Budaj A., Davies R.Y., Dellborg M., Fox K.A., Gutierrez J.A. (2016). Effect of losmapimod on cardiovascular outcomes in patients hospitalized with acute myocardial infarction: A randomized clinical trial. JAMA.

[11] Rymer J.A., Newby L.K. (2017). Failure to launch: Targeting inflammation in acute coronary syndromes. Basic Transl. Sci..

[12] Vallejo J., Cochain C., Zernecke A., Ley K. (2021). Heterogeneity of immune cells in human atherosclerosis revealed by scRNA-Seq. Cardiovasc. Res..

[13] de Winther M.P.J., Bäck M., Evans P., Gomez D., Goncalves I., Jørgensen H.F., Koenen R.R., Lutgens E., Norata G.D., Osto E. (2023). Translational opportunities of single-cell biology in atherosclerosis. Eur. Heart J..

[14] Milutinović A., Šuput D., Zorc-Pleskovič R. (2020). Pathogenesis of atherosclerosis in the tunica intima, media, and adventitia of coronary arteries: An updated review. Bosn. J. Basic Med. Sci..

[15] Tricot O., Mallat Z., Heymes C., Belmin J., Leseche G., Tedgui A. (2000). Relation between endothelial cell apoptosis and blood flow direction in human atherosclerotic plaques. Circulation.

[16] Zhang Y., Xie Y., You S., Han Q., Cao Y., Zhang X., Xiao G., Chen R., Liu C. (2015). Autophagy and apoptosis in the response of human vascular endothelial cells to oxidized low-density lipoprotein. Cardiology.

[17] Lee C.N., Cheng W.F., Chang M.C., Su Y.N., Chen C.A., Hsieh F.J. (2005). Hypoxia-induced apoptosis in endothelial cells and embryonic stem cells. Apoptosis.

[18] Rastogi S., Rizwani W., Joshi B., Kunigal S., Chellappan S.P. (2012). TNF-α response of vascular endothelial and vascular smooth muscle cells involve differential utilization of ASK1 kinase and p73. Cell Death Differ..

[19] Zhang J., Wang Z., Zuo G., Li B., Tian N., Chen S. (2013). Low shear stress induces human vascular endothelial cell apoptosis by activating Akt signal and increasing reactive oxygen species. J. South. Med. Univ..

[20] Dong G., Yang S., Cao X., Yu N., Yu J., Qu X. (2017). Low shear stress-induced autophagy alleviates cell apoptosis in HUVECs. Mol. Med. Rep..

[21] Hu Y.L., Hur S.S., Lei L., Wang Y., Chien S. (2017). Shear stress induces apoptosis via cytochrome c release from dynamic mitochondria in endothelial cells. FASEB J..

[22] Jimenez J.J., Jy W., Mauro L.M., Soderland C., Horstman L.L., Ahn Y.S. (2003). Endothelial cells release phenotypically and quantitatively distinct microparticles in activation and apoptosis. Thromb. Res..

[23] Abid Hussein M.N., Böing A.N., Biró E., Hoek F.J., Vogel G.M., Meuleman D.G., Sturk A., Nieuwland R. (2008). Phospholipid composition of in vitro endothelial microparticles and their in vivo thrombogenic properties. Thromb. Res..

[24] Mallat Z., Benamer H., Hugel B., Benessiano J., Steg P.G., Freyssinet J.M., Tedgui A. (2000). Elevated levels of shed membrane microparticles with procoagulant potential in the peripheral circulating blood of patients with acute coronary syndromes. Circulation.

[25] Bernal-Mizrachi L., Jy W., Jimenez J.J., Pastor J., Mauro L.M., Horstman L.L., de Marchena E., Ahn Y.S. (2003). High levels of circulating endothelial microparticles in patients with acute coronary syndromes. Am. Heart J..

[26] Alvandi Z., Bischoff J. (2021). Endothelial-mesenchymal transition in cardiovascular disease. Arterioscler. Thromb. Vasc. Biol..

[27] Evrard S.M., Lecce L., Michelis K.C., Nomura-Kitabayashi A., Pandey G., Purushothaman K.R., d’Escamard V., Li J.R., Hadri L., Fujitani K. (2016). Endothelial to mesenchymal transition is common in atherosclerotic lesions and is associated with plaque instability. Nat. Commun..

[28] Newman A.A.C., Serbulea V., Baylis R.A., Shankman L.S., Bradley X., Alencar G.F., Owsiany K., Deaton R.A., Karnewar S., Shamsuzzaman S. (2021). Multiple cell types contribute to the atherosclerotic lesion fibrous cap by PDGFRβ and bioenergetic mechanisms. Nat. Metab..

[29] Kovacic J.C., Dimmeler S., Harvey R.P., Finkel T., Aikawa E., Krenning G., Baker A.H. (2019). Endothelial to mesenchymal transition in cardiovascular disease: JACC state-of-the-art review. J. Am. Coll. Cardiol..

[30] Harman J.L., Jørgensen H.F. (2019). The role of smooth muscle cells in plaque stability: Therapeutic targeting potential. Br. J. Pharmacol..

[31] Burke A.P., Farb A., Malcom G.T., Liang Y.H., Smialek J., Virmani R. (1997). Coronary risk factors and plaque morphology in men with coronary disease who died suddenly. N. Engl. J. Med..

[32] Mocci G., Sukhavasi K., Örd T., Bankier S., Singha P., Arasu U.T., Agbabiaje O.O., Mäkinen P., Ma L., Hodonsky C.J. (2024). Single-cell gene-regulatory networks of advanced symptomatic atherosclerosis. Circ. Res..

[33] Basatemur G.L., Jørgensen H.F., Clarke M.C., Bennett M.R., Mallat Z. (2019). Vascular smooth muscle cells in atherosclerosis. Nat. Rev. Cardiol..

[34] Geng Y.J., Libby P. (1995). Evidence for apoptosis in advanced human atheroma. Colocalization with interleukin-1 beta-converting enzyme. Am. J. Pathol..

[35] Lutgens E., De Muinck E.D., Kitslaar P.J., Tordoir J.H., Wellens H.J., Daemen M.J. (1999). Biphasic pattern of cell turnover characterizes the progression from fatty streaks to ruptured human atherosclerotic plaques. Cardiovasc. Res..

[36] Kockx M.M., De Meyer G.R., Muhring J., Jacob W., Bult H., Herman A.G. (1998). Apoptosis and related proteins in different stages of human atherosclerotic plaques. Circulation.

[37] Clarke M.C., Figg N., Maguire J.J., Davenport A.P., Goddard M., Littlewood T.D., Bennett M.R. (2006). Apoptosis of vascular smooth muscle cells induces features of plaque vulnerability in atherosclerosis. Nat. Med..

[38] Clarke M.C., Littlewood T.D., Figg N., Maguire J.J., Davenport A.P., Goddard M., Bennett M.R. (2008). Chronic apoptosis of vascular smooth muscle cells accelerates atherosclerosis and promotes calcification and medial degeneration. Circ. Res..

[39] Bauriedel G., Hutter R., Welsch U., Bach R., Sievert H., Lüderitz B. (1999). Role of smooth muscle cell death in advanced coronary primary lesions: Implications for plaque instability. Cardiovasc. Res..

[40] Schrijvers D.M., De Meyer G.R., Kockx M.M., Herman A.G., Martinet W. (2005). Phagocytosis of apoptotic cells by macrophages is impaired in atherosclerosis. Arterioscler. Thromb. Vasc. Biol..

[41] Allahverdian S., Chehroudi A.C., McManus B.M., Abraham T., Francis G.A. (2014). Contribution of intimal smooth muscle cells to cholesterol accumulation and macrophage-like cells in human atherosclerosis. Circulation.

[42] Allahverdian S., Pannu P.S., Francis G.A. (2012). Contribution of monocyte-derived macrophages and smooth muscle cells to arterial foam cell formation. Cardiovasc. Res..

[43] Yancey P.G., Blakemore J., Ding L., Fan D., Overton C.D., Zhang Y., Linton M.F., Fazio S. (2010). Macrophage LRP-1 controls plaque cellularity by regulating efferocytosis and Akt activation. Arterioscler. Thromb. Vasc. Biol..

[44] Clarke M.C., Talib S., Figg N.L., Bennett M.R. (2010). Vascular smooth muscle cell apoptosis induces interleukin-1–directed inflammation: Effects of hyperlipidemia-mediated inhibition of phagocytosis. Circ. Res..

[45] Matthews C., Gorenne I., Scott S., Figg N., Kirkpatrick P., Ritchie A., Goddard M., Bennett M. (2006). Vascular smooth muscle cells undergo telomere-based senescence in human atherosclerosis: Effects of telomerase and oxidative stress. Circ. Res..

[46] Bennett M.R., Sinha S., Owens G.K. (2016). Vascular smooth muscle cells in atherosclerosis. Circ. Res..

[47] Wang J., Uryga A.K., Reinhold J., Figg N., Baker L., Finigan A., Gray K., Kumar S., Clarke M., Bennett M. (2015). Vascular smooth muscle cell senescence promotes atherosclerosis and features of plaque vulnerability. Circulation.

[48] Zhang X., Kang Z., Yin D., Gao J. (2023). Role of neutrophils in different stages of atherosclerosis. Innate Immun..

[49] Naruko T., Ueda M., Haze K., van der Wal A.C., van der Loos C.M., Itoh A., Komatsu R., Ikura Y., Ogami M., Shimada Y. (2002). Neutrophil infiltration of culprit lesions in acute coronary syndromes. Circulation.

[50] Soehnlein O. (2012). Multiple roles for neutrophils in atherosclerosis. Circ. Res..

[51] Quinn K.L., Henriques M., Tabuchi A., Han B., Yang H., Cheng W.E., Tole S., Yu H., Luo A., Charbonney E. (2011). Human neutrophil peptides mediate endothelial-monocyte interaction, foam cell formation, and platelet activation. Arterioscler. Thromb. Vasc. Biol..

[52] Grechowa I., Horke S., Wallrath A., Vahl C.F., Dorweiler B. (2017). Human neutrophil elastase induces endothelial cell apoptosis by activating the PERK-CHOP branch of the unfolded protein response. FASEB J..

[53] Moilanen M., Sorsa T., Stenman M., Nyberg P., Lindy O., Vesterinen J., Paju A., Konttinen Y.T., Stenman U.H., Salo T. (2003). Tumor-associated trypsinogen-2 (trypsinogen-2) activates procollagenases (MMP-1,-8,-13) and stromelysin-1 (MMP-3) and degrades type I collagen. Biochemistry.

[54] Momiyama Y., Ohmori R., Tanaka N., Kato R., Taniguchi H., Adachi T., Nakamura H., Ohsuzu F. (2010). High plasma levels of matrix metalloproteinase-8 in patients with unstable angina. Atherosclerosis.

[55] Herman M.P., Sukhova G.K., Libby P., Gerdes N., Tang N., Horton D.B., Kilbride M., Breitbart R.E., Chun M., Schönbeck U. (2001). Expression of neutrophil collagenase (matrix metalloproteinase-8) in human atheroma: A novel collagenolytic pathway suggested by transcriptional profiling. Circulation.

[56] Weiss S.J., Peppin G., Ortiz X., Ragsdale C., Test S.T. (1985). Oxidative autoactivation of latent collagenase by human neutrophils. Science.

[57] Dollery C.M., Owen C.A., Sukhova G.K., Krettek A., Shapiro S.D., Libby P. (2003). Neutrophil elastase in human atherosclerotic plaques: Production by macrophages. Circulation.

[58] Mokry M., Boltjes A., Slenders L., Bel-Bordes G., Cui K., Brouwer E., Mekke J.M., Depuydt M.A.C., Timmerman N., Waissi F. (2022). Transcriptomic-based clustering of human atherosclerotic plaques identifies subgroups with different underlying biology and clinical presentation. Nat. Cardiovasc. Res..

[59] Gierlikowska B., Stachura A., Gierlikowski W., Demkow U. (2021). Phagocytosis, degranulation and extracellular traps release by neutrophils—The current knowledge, pharmacological modulation and future prospects. Front. Pharmacol..

[60] Pertiwi K.R., van der Wal A.C., Pabittei D.R., Mackaaij C., van Leeuwen M.B., Li X., de Boer O.J. (2018). Neutrophil extracellular traps participate in all different types of thrombotic and haemorrhagic complications of coronary atherosclerosis. Thromb. Haemost..

[61] Quillard T., Araújo H.A., Franck G., Shvartz E., Sukhova G., Libby P. (2015). TLR2 and neutrophils potentiate endothelial stress, apoptosis and detachment: Implications for superficial erosion. Eur. Heart J..

[62] Saffarzadeh M., Juenemann C., Queisser M.A., Lochnit G., Barreto G., Galuska S.P., Lohmeyer J., Preissner K.T. (2012). Neutrophil extracellular traps directly induce epithelial and endothelial cell death: A predominant role of histones. PLoS ONE.

[63] Silvestre-Roig C., Braster Q., Wichapong K., Lee E.Y., Teulon J.M., Berrebeh N., Winter J., Adrover J.M., Santos G.S., Froese A. (2019). Externalized histone H4 orchestrates chronic inflammation by inducing lytic cell death. Nature.

[64] Kolodgie F.D., Narula J., Burke A.P., Haider N., Farb A., Hui-Liang Y., Smialek J., Virmani R. (2000). Localization of apoptotic macrophages at the site of plaque rupture in sudden coronary death. Am. J. Pathol..

[65] Popa-Fotea N.M., Ferdoschi C.E., Micheu M.M. (2023). Molecular and cellular mechanisms of inflammation in atherosclerosis. Front. Cardiovasc. Med..

[66] Gordon S. (2003). Alternative activation of macrophages. Nat. Rev. Immunol..

[67] Mallat Z., Hugel B., Ohan J., Leseche G., Freyssinet J.M., Tedgui A. (1999). Shed membrane microparticles with procoagulant potential in human atherosclerotic plaques: A role for apoptosis in plaque thrombogenicity. Circulation.

[68] Gonzalez L., Trigatti B.L. (2017). Macrophage apoptosis and necrotic core development in atherosclerosis: A rapidly advancing field with clinical relevance to imaging and therapy. Can. J. Cardiol..

[69] Thorp E., Tabas I. (2009). Mechanisms and consequences of efferocytosis in advanced atherosclerosis. J. Leukoc. Biol..

[70] Otsuka F., Kramer M.C., Woudstra P., Yahagi K., Ladich E., Finn A.V., de Winter R.J., Kolodgie F.D., Wight T.N., Davis H.R. (2015). Natural progression of atherosclerosis from pathologic intimal thickening to late fibroatheroma in human coronary arteries: A pathology study. Atherosclerosis.

[71] Björkegren J.L., Lusis A.J. (2022). Atherosclerosis: Recent developments. Cell.

[72] Huang W.C., Sala-Newby G.B., Susana A., Johnson J.L., Newby A.C. (2012). Classical macrophage activation up-regulates several matrix metalloproteinases through mitogen activated protein kinases and nuclear factor-κB. PLoS ONE.

[73] Lenglet S., Mach F., Montecucco F. (2013). Role of matrix metalloproteinase-8 in atherosclerosis. Mediat. Inflamm..

[74] Fligiel S.E., Varani J., Datta S.C., Kang S., Fisher G.J., Voorhees J.J. (2003). Collagen degradation in aged/photodamaged skin in vivo and after exposure to matrix metalloproteinase-1 in vitro. J. Investig. Dermatol..

[75] Sukhova G.K., Schönbeck U., Rabkin E., Schoen F.J., Poole A.R., Billinghurst R.C., Libby P. (1999). Evidence for increased collagenolysis by interstitial collagenases-1 and-3 in vulnerable human atheromatous plaques. Circulation.

[76] Shah P.K., Falk E., Badimon J.J., Fernandez-Ortiz A., Mailhac A., Villareal-Levy G., Fallon J.T., Regnstrom J., Fuster V. (1995). Human monocyte-derived macrophages induce collagen breakdown in fibrous caps of atherosclerotic plaques. Potential role of matrix-degrading metalloproteinases and implications for plaque rupture. Circulation.

[77] Shi X., Xie W.L., Kong W.W., Chen D., Qu P. (2015). Expression of the NLRP3 inflammasome in carotid atherosclerosis. J. Stroke Cerebrovasc. Dis..

[78] Silvis M.J.M., Demkes E.J., Fiolet A.T.L., Dekker M., Bosch L., van Hout G.P.J., Timmers L., de Kleijn D.P.V. (2021). Immunomodulation of the NLRP3 inflammasome in atherosclerosis, coronary artery disease, and acute myocardial infarction. J. Cardiovasc. Transl. Res..

[79] Kong P., Cui Z.Y., Huang X.F., Zhang D.D., Guo R.J., Han M. (2022). Inflammation and atherosclerosis: Signaling pathways and therapeutic intervention. Signal Transduct. Target. Ther..

[80] Misawa T., Takahama M., Kozaki T., Lee H., Zou J., Saitoh T., Akira S. (2013). Microtubule-driven spatial arrangement of mitochondria promotes activation of the NLRP3 inflammasome. Nat. Immunol..

[81] Paramel Varghese G., Folkersen L., Strawbridge R.J., Halvorsen B., Yndestad A., Ranheim T., Krohg-Sørensen K., Skjelland M., Espevik T., Aukrust P. (2016). NLRP 3 inflammasome expression and activation in human atherosclerosis. J. Am. Heart Assoc..

[82] Jiang C., Jiang L., Li Q., Liu X., Zhang T., Dong L., Liu T., Liu L., Hu G., Sun X. (2018). Acrolein induces NLRP3 inflammasome-mediated pyroptosis and suppresses migration via ROS-dependent autophagy in vascular endothelial cells. Toxicology.

[83] Burger F., Baptista D., Roth A., da Silva R.F., Montecucco F., Mach F., Brandt K.J., Miteva K. (2021). NLRP3 inflammasome activation controls vascular smooth muscle cells phenotypic switch in atherosclerosis. Int. J. Mol. Sci..

[84] Münzer P., Negro R., Fukui S., di Meglio L., Aymonnier K., Chu L., Cherpokova D., Gutch S., Sorvillo N., Shi L. (2021). NLRP3 inflammasome assembly in neutrophils is supported by PAD4 and promotes NETosis under sterile conditions. Front. Immunol..

[85] Zheng F., Xing S., Gong Z., Mu W., Xing Q. (2014). Silence of NLRP3 suppresses atherosclerosis and stabilizes plaques in apolipoprotein E-deficient mice. Mediat. Inflamm..

[86] Jin M., Fang J., Wang J.J., Shao X., Xu S.W., Liu P.Q., Ye W.C., Liu Z.P. (2023). Regulation of toll-like receptor (TLR) signaling pathways in atherosclerosis: From mechanisms to targeted therapeutics. Acta Pharmacol. Sin..

[87] Monaco C., Gregan S.M., Navin T.J., Foxwell B.M., Davies A.H., Feldmann M. (2009). Toll-like receptor-2 mediates inflammation and matrix degradation in human atherosclerosis. Circulation.

[88] Edfeldt K., Swedenborg J., Hansson G.K., Yan Z.Q. (2002). Expression of toll-like receptors in human atherosclerotic lesions: A possible pathway for plaque activation. Circulation.

[89] Yang K., Zhang X.J., Cao L.J., Liu X.H., Liu Z.H., Wang X.Q., Chen Q.J., Lu L., Shen W.F., Liu Y. (2014). Toll-like receptor 4 mediates inflammatory cytokine secretion in smooth muscle cells induced by oxidized low-density lipoprotein. PLoS ONE.

[90] Koushki K., Shahbaz S.K., Mashayekhi K., Sadeghi M., Zayeri Z.D., Taba M.Y., Banach M., Al-Rasadi K., Johnston T.P., Sahebkar A. (2021). Anti-inflammatory action of statins in cardiovascular disease: The role of inflammasome and toll-like receptor pathways. Clin. Rev. Allergy Immunol..

[91] Wang L., Peng Y., Song L., Xia D., Li C., Li Z., Li Q., Yu A., Lu C., Wang Y. (2022). Colchicine-containing nanoparticles attenuates acute myocardial infarction injury by inhibiting inflammation. Cardiovasc. Drugs Ther..

[92] Kiss M.G., Binder C.J. (2022). The multifaceted impact of complement on atherosclerosis. Atherosclerosis.

[93] Martínez-López D., Roldan-Montero R., García-Marqués F., Nuñez E., Jorge I., Camafeita E., Minguez P., Rodriguez de Cordoba S., López-Melgar B., Lara-Pezzi E. (2020). Complement C5 protein as a marker of subclinical atherosclerosis. J. Am. Coll. Cardiol..

[94] Garcia-Arguinzonis M., Diaz-Riera E., Peña E., Escate R., Juan-Babot O., Mata P., Badimon L., Padro T. (2021). Alternative C3 complement system: Lipids and atherosclerosis. Int. J. Mol. Sci..

[95] Oksjoki R., Laine P., Helske S., Vehmaan-Kreula P., Mäyränpää M.I., Gasque P., Kovanen P.T., Pentikäinen M.O. (2007). Receptors for the anaphylatoxins C3a and C5a are expressed in human atherosclerotic coronary plaques. Atherosclerosis.

[96] Speidl W.S., Kastl S.P., Hutter R., Katsaros K.M., Kaun C., Bauriedel G., Maurer G., Huber K., Badimon J.J., Wojta J. (2011). The complement component C5a is present in human coronary lesions in vivo and induces the expression of MMP-1 and MMP-9 in human macrophages in vitro. FASEB J..

[97] Speidl W.S., Exner M., Amighi J., Kastl S.P., Zorn G., Maurer G., Wagner O., Huber K., Minar E., Wojta J. (2005). Complement component C5a predicts future cardiovascular events in patients with advanced atherosclerosis. Eur. Heart J..

[98] Oksjoki R., Kovanen P.T., Mäyränpää M.I., Laine P., Blom A.M., Meri S., Pentikäinen M.O. (2007). Complement regulation in human atherosclerotic coronary lesions: Immunohistochemical evidence that C4b-binding protein negatively regulates the classical complement pathway, and that C5b-9 is formed via the alternative complement pathway. Atherosclerosis.

[99] Si W., He P., Wang Y., Fu Y., Li X., Lin X., Chen F., Cao G., Zhang H. (2019). Complement complex C5b-9 levels are associated with the clinical outcomes of acute ischemic stroke and carotid plaque stability. Transl. Stroke Res..

[100] Lindberg S., Pedersen S.H., Mogelvang R., Galatius S., Flyvbjerg A., Jensen J.S., Bjerre M. (2012). Soluble form of membrane attack complex independently predicts mortality and cardiovascular events in patients with ST-elevation myocardial infarction treated with primary percutaneous coronary intervention. Am. Heart J..

[101] Monsinjon T., Gasque P., Chan P., Ischenko A., Brady J.J., Fontaine M. (2003). Regulation by complement C3a and C5a anaphylatoxins of cytokine production in human umbilical vein endothelial cells. FASEB J..

[102] Samstad E.O., Niyonzima N., Nymo S., Aune M.H., Ryan L., Bakke S.S., Lappegård K.T., Brekke O.L., Lambris J.D., Damås J.K. (2014). Cholesterol crystals induce complement-dependent inflammasome activation and cytokine release. J. Immunol..

[103] Niyonzima N., Bakke S.S., Gregersen I., Holm S., Sandanger Ø., Orrem H.L., Sporsheim B., Ryan L., Kong X.Y., Dahl T.B. (2020). Cholesterol crystals use complement to increase NLRP3 signaling pathways in coronary and carotid atherosclerosis. EBioMedicine.

[104] Ikeda K., Nagasawa K., Horiuchi T., Tsuru T., Nishizaka H., Niho Y. (1997). C5a induces tissue factor activity on endothelial cells. Thromb. Haemost..

[105] Li B., Xu Y.J., Chu X.M., Gao M.H., Wang X.H., Nie S.M., Yang F., Lv C.Y. (2013). Molecular mechanism of inhibitory effects of CD59 gene on atherosclerosis in *ApoE* (−/−) mice. Immunol. Lett..

[106] Fosbrink M., Niculescu F., Rus H. (2005). The role of c5b-9 terminal complement complex in activation of the cell cycle and transcription. Immunol. Res..

[107] Pulanco M.C., Cosman J., Ho M.M., Huynh J., Fing K., Turcu J., Fraser D.A. (2017). Complement protein C1q enhances macrophage foam cell survival and efferocytosis. J. Immunol..

[108] Wei L.L., Ma N., Wu K.Y., Wang J.X., Diao T.Y., Zhao S.J., Bai L., Liu E., Li Z.F., Zhou W. (2020). Protective role of C3aR (C3a anaphylatoxin receptor) against atherosclerosis in atherosclerosis-prone mice. Arterioscler. Thromb. Vasc. Biol..

[109] Pang Q., You L., Meng X., Li Y., Deng T., Li D., Zhu B. (2023). Regulation of the JAK/STAT signaling pathway: The promising targets for cardiovascular disease. Biochem. Pharmacol..

[110] Chen Q., Lv J., Yang W., Xu B., Wang Z., Yu Z., Wu J., Yang Y., Han Y. (2019). Targeted inhibition of STAT3 as a potential treatment strategy for atherosclerosis. Theranostics.

[111] Zhang X., Chen S., Yin G., Liang P., Feng Y., Yu W., Meng D., Liu H., Zhang F. (2024). The Role of JAK/STAT Signaling Pathway and Its Downstream Influencing Factors in the Treatment of Atherosclerosis. J. Cardiovasc. Pharmacol. Ther..

[112] Mazière C., Conte M.A., Mazière J.C. (2001). Activation of JAK2 by the oxidative stress generated with oxidized low-density lipoprotein. Free Radic. Biol. Med..

[113] Taleb S., Romain M., Ramkhelawon B., Uyttenhove C., Pasterkamp G., Herbin O., Esposito B., Perez N., Yasukawa H., Van Snick J. (2009). Loss of SOCS3 expression in T cells reveals a regulatory role for interleukin-17 in atherosclerosis. J. Exp. Med..

[114] Gharavi N.M., Alva J.A., Mouillesseaux K.P., Lai C., Yeh M., Yeung W., Johnson J., Szeto W.L., Hong L., Fishbein M. (2007). Role of the Jak/STAT pathway in the regulation of interleukin-8 transcription by oxidized phospholipids in vitro and in atherosclerosis in vivo. J. Biol. Chem..

[115] Manea A., Tanase L.I., Raicu M., Simionescu M. (2010). Jak/STAT signaling pathway regulates nox1 and nox4-based NADPH oxidase in human aortic smooth muscle cells. Arterioscler. Thromb. Vasc. Biol..

[116] Yeh M., Gharavi N.M., Choi J., Hsieh X., Reed E., Mouillesseaux K.P., Cole A.L., Reddy S.T., Berliner J.A. (2004). Oxidized phospholipids increase interleukin 8 (IL-8) synthesis by activation of the c-src/signal transducers and activators of transcription (STAT) 3 pathway. J. Biol. Chem..

[117] Alanazi A.Z., Clark M.A. (2019). Angiotensin III induces JAK2/STAT3 leading to IL-6 production in rat vascular smooth muscle cells. Int. J. Mol. Sci..

[118] Khan J.A., Cao M., Kang B.Y., Liu Y., Mehta J.L., Hermonat P.L. (2010). AAV/hSTAT3-gene delivery lowers aortic inflammatory cell infiltration in LDLR KO mice on high cholesterol. Atherosclerosis.

[119] Wang K., Li B., Xie Y., Xia N., Li M., Gao G. (2020). Statin rosuvastatin inhibits apoptosis of human coronary artery endothelial cells through upregulation of the JAK2/STAT3 signaling pathway. Mol. Med. Rep..

[120] Dutzmann J., Daniel J.M., Bauersachs J., Hilfiker-Kleiner D., Sedding D.G. (2015). Emerging translational approaches to target STAT3 signalling and its impact on vascular disease. Cardiovasc. Res..

[121] Dinarello C.A. (2018). Overview of the IL-1 family in innate inflammation and acquired immunity. Immunol. Rev..

[122] Thompson P.L., Nidorf S.M. (2018). Anti-inflammatory therapy with canakinumab for atherosclerotic disease: Lessons from the CANTOS trial. J. Thorac. Dis..

[123] Abbate A., Toldo S., Marchetti C., Kron J., Van Tassell B.W., Dinarello C.A. (2020). Interleukin-1 and the inflammasome as therapeutic targets in cardiovascular disease. Circ. Res..

[124] Ridker P.M., Everett B.M., Thuren T., MacFadyen J.G., Chang W.H., Ballantyne C., Fonseca F., Nicolau J., Koenig W., Anker S.D. (2017). Antiinflammatory therapy with canakinumab for atherosclerotic disease. N. Engl. J. Med..

[125] Morton A.C., Rothman A.M., Greenwood J.P., Gunn J., Chase A., Clarke B., Hall A.S., Fox K., Foley C., Banya W. (2015). The effect of interleukin-1 receptor antagonist therapy on markers of inflammation in non-ST elevation acute coronary syndromes: The MRC-ILA Heart Study. Eur. Heart J..

[126] Abbate A., Trankle C.R., Buckley L.F., Lipinski M.J., Appleton D., Kadariya D., Canada J.M., Carbone S., Roberts C.S., Abouzaki N. (2020). Interleukin-1 blockade inhibits the acute inflammatory response in patients with ST-segment–elevation myocardial infarction. J. Am. Heart Assoc..

[127] Van Tassell B.W., Toldo S., Mezzaroma E., Abbate A. (2013). Targeting interleukin-1 in heart disease. Circulation.

[128] Interleukin-1 Blockade in Acute Myocardial Infarction to Prevent Heart Failure (VA-ART4). Clinicaltrials.gov. https://clinicaltrials.gov/study/NCT05177822.

[129] Anderson D.R., Poterucha J.T., Mikuls T.R., Duryee M.J., Garvin R.P., Klassen L.W., Shurmur S.W., Thiele G.M. (2013). IL-6 and its receptors in coronary artery disease and acute myocardial infarction. Cytokine.

[130] Peeters W., Hellings W.E., De Kleijn D.P.V., De Vries J.P.P.M., Moll F.L., Vink A., Pasterkamp G. (2009). Carotid atherosclerotic plaques stabilize after stroke: Insights into the natural process of atherosclerotic plaque stabilization. Arterioscler. Thromb. Vasc. Biol..

[131] Reiss A.B., Siegart N.M., De Leon J. (2017). Interleukin-6 in atherosclerosis: Atherogenic or atheroprotective?. Clin. Lipidol..

[132] Garbers C., Aparicio-Siegmund S., Rose-John S. (2015). The IL-6/gp130/STAT3 signaling axis: Recent advances towards specific inhibition. Curr. Opin. Immunol..

[133] Rakocevic J., Dobric M., Borovic M.L., Milutinovic K., Milenkovic S., Tomasevic M. (2023). Anti-inflammatory therapy in coronary artery disease: Where do we stand?. Rev. Cardiovasc. Med..

[134] Kleveland O., Kunszt G., Bratlie M., Ueland T., Broch K., Holte E., Michelsen A.E., Bendz B., Amundsen B.H., Espevik T. (2016). Effect of a single dose of the interleukin-6 receptor antagonist tocilizumab on inflammation and troponin T release in patients with non-ST-elevation myocardial infarction: A double-blind, randomized, placebo-controlled phase 2 trial. Eur. Heart J..

[135] Broch K., Anstensrud A.K., Woxholt S., Sharma K., Tøllefsen I.M., Bendz B., Aakhus S., Ueland T., Amundsen B.H., Damås J.K. (2021). Randomized trial of interleukin-6 receptor inhibition in patients with acute ST-segment elevation myocardial infarction. J. Am. Coll. Cardiol..

[136] Holle S.L.D., Kunkel J.B., Hassager C., Pecini R., Wiberg S., Palm P., Holmvang L., Bang L.E., Kjærgaard J., Thomsen J.H. (2023). Low-dose dobutamine infusion and single-dose tocilizumab in acute myocardial infarction patients with high risk of cardiogenic shock development-rationale and design of the DOBERMANN trial. Eur. Heart J. Acute Cardiovasc. Care.

[137] Kawashiri S.Y., Kawakami A., Yamasaki S., Imazato T., Iwamoto N., Fujikawa K., Aramaki T., Tamai M., Nakamura H., Ida H. (2011). Effects of the anti-interleukin-6 receptor antibody, tocilizumab, on serum lipid levels in patients with rheumatoid arthritis. Rheumatol. Int..

[138] Ridker P.M. (2021). From RESCUE to ZEUS: Will interleukin-6 inhibition with ziltivekimab prove effective for cardiovascular event reduction?. Cardiovasc. Res..

[139] Specifying the Anti-inflammatory Effects of Ziltivekimab (SPIDER). Clinicaltrials.gov. https://clinicaltrials.gov/study/NCT06263244.

[140] ARTEMIS—A Research Study to Look at How Ziltivekimab Works Compared to Placebo in People With a Heart Attack (ARTEMIS). Clinicaltrials.gov. https://clinicaltrials.gov/study/NCT06118281.

[141] Mann D.L., McMurray J.J., Packer M., Swedberg K., Borer J.S., Colucci W.S., Djian J., Drexler H., Feldman A., Kober L. (2004). Targeted anticytokine therapy in patients with chronic heart failure: Results of the Randomized Etanercept Worldwide Evaluation (RENEWAL). Circulation.

[142] Gonzalez-Juanatey C., Vazquez-Rodriguez T.R., Miranda-Filloy J.A., Gomez-Acebo I., Testa A., Garcia-Porrua C., Sanchez-Andrade A., Llorca J., González-Gay M.A. (2012). Anti-TNF-alpha-adalimumab therapy is associated with persistent improvement of endothelial function without progression of carotid intima-media wall thickness in patients with rheumatoid arthritis refractory to conventional therapy. Mediat. Inflamm..

[143] Kearney N., Chen X., Bi Y., Hew K., Smith K.M., Kirby B. (2024). Treatment of hidradenitis suppurativa with adalimumab in the PIONEER I and II randomized controlled trials reduced indices of systemic inflammation, recognized risk factors for cardiovascular disease. Clin. Exp. Dermatol..

[144] Elkhawad M., Rudd J.H., Sarov-Blat L., Cai G., Wells R., Davies L.C., Collier D.J., Marber M.S., Choudhury R.P., Fayad Z.A. (2012). Effects of p38 mitogen-activated protein kinase inhibition on vascular and systemic inflammation in patients with atherosclerosis. JACC Cardiovasc. Imaging.

[145] Newby L.K., Marber M.S., Melloni C., Sarov-Blat L., Aberle L.H., Aylward P.E., Cai G., de Winter R.J., Hamm C.W., Heitner J.F. (2014). Losmapimod, a novel p38 mitogen-activated protein kinase inhibitor, in non-ST-segment elevation myocardial infarction: A randomised phase 2 trial. Lancet.

[146] Sarov-Blat L., Morgan J.M., Fernandez P., James R., Fang Z., Hurle M.R., Baidoo C., Willette R.N., Lepore J.J., Jensen S.E. (2010). Inhibition of p38 mitogen-activated protein kinase reduces inflammation after coronary vascular injury in humans. Arterioscler. Thromb. Vasc. Biol..

[147] Leung Y.Y., Hui L.L.Y., Kraus V.B. (2015). Colchicine—Update on mechanisms of action and therapeutic uses. Semin. Arthritis Rheum..

[148] Forkosh E., Kenig A., Ilan Y. (2020). Introducing variability in targeting the microtubules: Review of current mechanisms and future directions in colchicine therapy. Pharmacol. Res. Perspect..

[149] Takenouchi T., Iwamaru Y., Sugama S., Sato M., Hashimoto M., Kitani H. (2008). Lysophospholipids and ATP mutually suppress maturation and release of IL-1β in mouse microglial cells using a Rho-dependent pathway. J. Immunol..

[150] Tardif J.C., Kouz S., Waters D.D., Bertrand O.F., Diaz R., Maggioni A.P., Pinto F.J., Ibrahim R., Gamra H., Kiwan G.S. (2019). Efficacy and safety of low-dose colchicine after myocardial infarction. N. Engl. J. Med..

[151] Nidorf S.M., Fiolet A.T.L., Mosterd A., Eikelboom J.W., Schut A., Opstal T.S.J., The S.H.K., Xu X.F., Ireland M.A., Lenderink T. (2020). Colchicine in patients with chronic coronary disease. N. Engl. J. Med..

[152] Tong D.C., Quinn S., Nasis A., Hiew C., Roberts-Thomson P., Adams H., Sriamareswaran R., Htun N.M., Wilson W., Stub D. (2020). Colchicine in patients with acute coronary syndrome: The Australian COPS randomized clinical trial. Circulation.

[153] D’Entremont M.A., Lee S.F., Mian R., Kedev S., Montalescot G., Cornel J.H., Stankovic G., Moreno R., Storey R.F., Henry T.D. (2024). Design and rationale of the CLEAR SYNERGY (OASIS 9) trial: A 2 × 2 factorial randomized controlled trial of colchicine versus placebo and spironolactone vs placebo in patients with myocardial infarction. Am. Heart J..

[154] Kojima Y., Volkmer J.P., McKenna K., Civelek M., Lusis A.J., Miller C.L., Direnzo D., Nanda V., Ye J., Connolly A.J. (2016). CD47-blocking antibodies restore phagocytosis and prevent atherosclerosis. Nature.

[155] Rolski F., Błyszczuk P. (2020). Complexity of TNF-α signaling in heart disease. J. Clin. Med..

[156] Brånén L., Hovgaard L., Nitulescu M., Bengtsson E., Nilsson J., Jovinge S. (2004). Inhibition of tumor necrosis factor-α reduces atherosclerosis in apolipoprotein E knockout mice. Arterioscler. Thromb. Vasc. Biol..

[157] Chistiakov D.A., Melnichenko A.A., Grechko A.V., Myasoedova V.A., Orekhov A.N. (2018). Potential of anti-inflammatory agents for treatment of atherosclerosis. Exp. Mol. Pathol..

[158] Oberoi R., Schuett J., Schuett H., Koch A.K., Luchtefeld M., Grote K., Schieffer B. (2016). Targeting tumor necrosis factor-α with adalimumab: Effects on endothelial activation and monocyte adhesion. PLoS ONE.

[159] Boesten L.S., Zadelaar A.S.M., van Nieuwkoop A., Gijbels M.J., de Winther M.P., Havekes L.M., van Vlijmen B.J. (2005). Tumor necrosis factor-α promotes atherosclerotic lesion progression in APOE* 3-leiden transgenic mice. Cardiovasc. Res..

[160] Fisk M., Gajendragadkar P.R., Mäki-Petäjä K.M., Wilkinson I.B., Cheriyan J. (2014). Therapeutic potential of p38 MAP kinase inhibition in the management of cardiovascular disease. Am. J. Cardiovasc. Drugs.

[161] Hammaker D., Firestein G.S. (2010). “Go upstream, young man”: Lessons learned from the p38 saga. Ann. Rheum. Dis..

[162] Vaidya K., Tucker B., Kurup R., Khandkar C., Pandzic E., Barraclough J., Machet J., Misra A., Kavurma M., Martinez G. (2021). Colchicine inhibits neutrophil extracellular trap formation in patients with acute coronary syndrome after percutaneous coronary intervention. J. Am. Heart Assoc..

[163] Diamantis E., Kyriakos G., Victoria Quiles-Sanchez L., Farmaki P., Troupis T. (2017). The anti-inflammatory effects of statins on coronary artery disease: An updated review of the literature. Curr. Cardiol. Rev..

[164] Ridker P.M., Danielson E., Fonseca F.A., Genest J., Gotto AMJr Kastelein J.J., Koenig W., Libby P., Lorenzatti A.J., MacFadyen J.G., Nordestgaard B.G. (2008). Rosuvastatin to prevent vascular events in men and women with elevated C-reactive protein. N. Engl. J. Med..

[165] Stefanadi E., Tousoulis D., Antoniades C., Katsi V., Bosinakou E., Vavuranakis E., Triantafyllou G., Marinou K., Tsioufis C., Papageorgiou N. (2009). Early initiation of low-dose atorvastatin treatment after an acute ST-elevated myocardial infarction, decreases inflammatory process and prevents endothelial injury and activation. Int. J. Cardiol..

[166] Chen J., Zhang X., Millican R., Lynd T., Gangasani M., Malhotra S., Sherwood J., Hwang P.T., Cho Y., Brott B.C. (2022). Recent progress in in vitro models for atherosclerosis studies. Front. Cardiovasc. Med..

[167] Zhang Y., Fatima M., Hou S., Bai L., Zhao S., Liu E. (2021). Research methods for animal models of atherosclerosis. Mol. Med. Rep..

[168] Seeger F.H., Sedding D., Langheinrich A.C., Haendeler J., Zeiher A.M., Dimmeler S. (2010). Inhibition of the p38 MAP kinase in vivo improves number and functional activity of vasculogenic cells and reduces atherosclerotic disease progression. Basic Res. Cardiol..

[169] Golforoush P., Yellon D.M., Davidson S.M. (2020). Mouse models of atherosclerosis and their suitability for the study of myocardial infarction. Basic Res. Cardiol..

[170] Han Y., Gao S., Muegge K., Zhang W., Zhou B. (2015). Advanced applications of RNA sequencing and challenges. Bioinform. Biol. Insights.

[171] Ramazi S., Zahiri J. (2021). Post-translational modifications in proteins: Resources, tools and prediction methods. Database.

[172] Baldini C., Moriconi F.R., Galimberti S., Libby P., De Caterina R. (2021). The JAK–STAT pathway: An emerging target for cardiovascular disease in rheumatoid arthritis and myeloproliferative neoplasms. Eur. Heart J..

[173] Schlotter F., Halu A., Goto S., Blaser M.C., Body S.C., Lee L.H., Higashi H., DeLaughter D.M., Hutcheson J.D., Vyas P. (2018). Spatiotemporal multi-omics mapping generates a molecular atlas of the aortic valve and reveals networks driving disease. Circulation.

